# PIPKI*γ* Regulates CCL2 Expression in Colorectal Cancer by Activating AKT-STAT3 Signaling

**DOI:** 10.1155/2019/3690561

**Published:** 2019-11-03

**Authors:** JunLi Xue, XiaoXiao Ge, Wei Zhao, Liqiong Xue, Congqi Dai, Fengjuan Lin, Wei Peng

**Affiliations:** Department of Oncology, Shanghai East Hospital, Tongji University School of Medicine, 1800 Yuntai Road, Pudong District, Shanghai 200123, China

## Abstract

Colorectal cancer (CRC) remains the third most commonly diagnosed cancer, ranking second among the most common causes of cancer-related mortality. Immune checkpoint therapy has recently been shown to have great potential. However, only some patients respond to immune checkpoint blockade, indicating the unmet need for determining the underlying mechanism of colorectal cancer immunosuppression. In this study, we analyzed The Cancer Genome Atlas (TCGA) datasets and found that high expression of PIPKI*γ* positively correlated with tumor-associated macrophage infiltration. Further loss-of-function studies revealed that silencing PIPKI*γ* greatly reduced CCL2 expression at both the mRNA and protein levels, leading to weak chemotaxis of cancer cells to macrophages. Mechanistically, PIPKI*γ* facilitated PI3K-Akt-mTOR signaling pathway activation to increase STAT3 phosphorylation levels, thus triggering *CCL2* transcription to enhance tumor-associated macrophage recruitment. These findings identify the PIPKI*γ* signaling pathway as a new actor in colorectal cancer immunosuppression and a potential therapeutic target for this common cancer.

## 1. Introduction

Colorectal cancer (CRC) is one of the most common malignant tumors of the digestive system. Currently, the incidence of colorectal cancer is ranked third among malignant tumors [1, 2]. In 2019, there were more than 130,000 new patients with colorectal cancer, and more than 50,000 people died of colorectal cancer in the United States. Worldwide, the incidence of colorectal cancer is also on the rise, which emphasizes the importance of further understanding the mechanisms of CRC initiation and progression.

Previous studies have reported that the interaction between tumor cells and the microenvironment, especially transformed cells and infiltrating immune cells, greatly supports the progression of cancer [3, 4]. Treatments such as PD-1/PD-L1 checkpoint blockade [5] and chemokine regulation have successfully altered the effects of the interaction between the immune system and cancer on rejection or, at least, have inhibited progression [6]. However, only 20%-30% of patients respond to immunological treatment [7]. Previous studies reported that cancer cells could reshape the immune microenvironment and the function of immune cells. The most important factor in this process is tumor-associated macrophages (TAMs), which originate mainly from monocytes that are recruited to the tumor microenvironment. TAMs could exert immunosuppressive effects by releasing cytokines/chemokines, expressing checkpoint ligands and inducing cytotoxic T cell apoptosis, leading to immunosuppression and immune evasion. These findings thus emphasize the importance of uncovering mechanisms of how cancer cells recruit and educate immune cells.

Type I*γ* phosphatidylinositol phosphate kinase (PIPKI*γ*), encoded by PIP5K1C, is a crucial enzyme that plays a key role in multiple biological processes by regulating PI4, 5P2 synthesis [8, 9]. PIPKI*γ* was reported to regulate cell migration in multiple ways, such as through the EGF receptor (EGFR), upon Y639 phosphorylation by receptor tyrosine kinases (RTKs) [10, 11]. PIPKI*γ* could regulate neoplastic adhesion formation at the front edge through direct interaction with talin [12]. Additionally, PIPKI*γ* could bind to AP2, an adaptor of E-cadherin to clathrin, to reform E-cadherin-based intercellular adhesions and restore epithelial polarization [13]. Indeed, recent work shows that upregulation of PIPKI*γ* expression inversely correlates with the overall survival of patients with various types of cancer [14, 15]. However, the roles of PIPKI*γ* in tumor immunosuppression microenvironment formation remain unclear.

In this study, we aimed to identify the relationship between PIPKI*γ* and the tumor immunosuppression microenvironment. By analyzing TCGA data, we found that PIPKI*γ* expression was positively correlated with macrophage infiltration. Mechanistically, high PIPKI*γ* expression in CRC cancer increased CCL2 expression by activating the AKT-STAT3 signaling axis, further facilitating macrophage infiltration.

## 2. Materials and Methods

### 2.1. Cell Lines

Colorectal cancer cell lines HCT116, SW620, LOVO, and SW480 were obtained from the American Type Culture Collection (ATCC) and were grown in regular DMEM (Dulbecco's modified Eagle's medium, Gibco) or RPMI 1640 medium (Gibco, USA) supplemented with 10% fetal bovine serum (FBS, Gibco, USA), 1% L-glutamine, and 1% penicillin/streptomycin. Cells were maintained in a humidified incubator with a 5% CO_2_ atmosphere. All cell lines were tested for mycoplasma using the MycoAlert mycoplasma detection kit (Lonza, Portsmouth, NH).

### 2.2. RNA Interference Studies

For shRNA-mediated knockdown of gene expression experiments, SW480 and LOVO cells were infected with the lentivirus of control (Ctrl), sh PIP5I*γ*-1, or sh PIP5I*γ*-2 for 48 h and treated with 2 *μ*g/ml puromycin for a further one week to select the stable clones. For siRNA-mediated genes knockdown, Lipofectamine RNAiMAX (Invitrogen) was used following the protocol provided by manufacturer, and cells were used 48–72 h posttransfection. Knockdown efficiency of PIPKI*γ* was confirmed by Q-PCR and Western blotting analysis.

### 2.3. ELISA

Colorectal cancer cells with the indicated treatment were washed twice with PBS and incubated with FBS-free medium for 24 h. Then, the cell medium was collected and centrifuged at 12,000 rpm at 4°C. The supernatants were collected and used immediately. CCL2, CCL5, and TGF*β*1 ELISA kits were purchased from BD Biosciences and used according to the manufacturer's protocol. Total cell protein was detected for supernatant normalization.

### 2.4. Luciferase Assay

293 T cells were plated at 60-70% confluence in 24-well plates and transiently transfected with 1 *μ*g of different fragments of CCL2 promoter-connected luciferase reporter constructs together with expression plasmids for STAT3 using FuGENE 6 transfection reagent (Roche Applied Science). Empty pcDNA3.1 vector was used as a control. Firefly luciferase activity was normalized to that of Renilla luciferase, which were cotransfected under the control of the SV40 early enhancer/promoter region (pSV40-RL, Promega).

### 2.5. Patients and Samples

Patients with CRC were from Shanghai East Hospital, School of Medicine, Tongji University. The study was conducted in accordance with International Ethical Guidelines for Biomedical Research Involving Human Subjects (CIOMS). The study was approved by the Research Ethics Committee of Shanghai East Hospital. Patient samples for a human CRC tissue array containing 75 CRC specimens were also obtained from Shanghai East Hospital. Written informed consent was provided to all the patients before enrollment. Patients who had not received radiotherapy, chemotherapy, or other related antitumor therapies before surgery were enrolled in this study.

### 2.6. Immunohistochemical Analysis

First, paraffin-embedded sections were deparaffinized in xylene and rehydrated with decreasing concentrations of ethanol. Next, the sections were boiled in 10 mM citrate buffer (pH 6.0) for 10 min for antigen retrieval, followed by 3% hydrogen peroxide treatment at 37°C for 30 min to block endogenous peroxidase activity. Then, 10% BSA was used to block the sections, and the sections were incubated with a primary antibody overnight at 4°C. The antibodies used for immunohistochemistry were as follows: PIPKI*γ* (1 : 200, Proteintech, 27640-1-AP), CD163 (1 : 300, Abcam, ab182422), and p-STAT3 (1 : 100, Cell Signaling Technology, #9145). Then, the corresponding HRP-conjugated secondary antibody was incubated with the slides for 1 h at room temperature. The reactions were visualized using 3,3′-diaminobenzidine (DAB). Finally, the sections were counterstained with hematoxylin.

### 2.7. Chromatin Immunoprecipitation (ChIP)

SW480 and LOVO cells were cross-linked with 1% formaldehyde at room temperature for 10 min. Cross-linking was then quenched with 0.125 M glycine at room temperature for 5 min, and the cells were washed twice with cold PBS and then scraped and sonicated in lysis buffer for 20 min. Chromatin was centrifuged out at 14,000 rpm for 15 min, and the supernatants were diluted 10-fold in dilution buffer. The supernatants were incubated with protein G magnetic beads (Millipore 16-662) and STAT3 antibody (1 : 100, Cell Signaling Technology, #9139) overnight at 4°C. The next day, the samples were washed with decreasingly stringent buffers 5 times. ChIP DNA was eluted from the beads with elution buffer for 10 min at 65°C. The supernatants were then incubated overnight with proteinase K. The DNA samples were purified using a Macherey-Nagel DNA purification kit for quantitative PCR, and the ChIP samples were diluted 1 : 20 and used as a template with Power Sybr Master Mix (ABI 4367659); DNA was amplified using a ViiA-7 Real-Time PCR system. The primer sequences are listed in Supplementary [Supplementary-material supplementary-material-1].

### 2.8. Statistics

All data are presented as the mean ± SD. All experiments were performed with a minimum of three independent replicates. Statistical analysis was performed using GraphPad Prism 5.0. Student's *t*-test or one-way ANOVA was used for comparisons between groups. In all tests, *p* values of less than 0.05 were considered statistically significant.

## 3. Results

### 3.1. Increased PIPKI*γ* Expression Positively Correlated with TAM Infiltration in CRC

**Figure 1:**
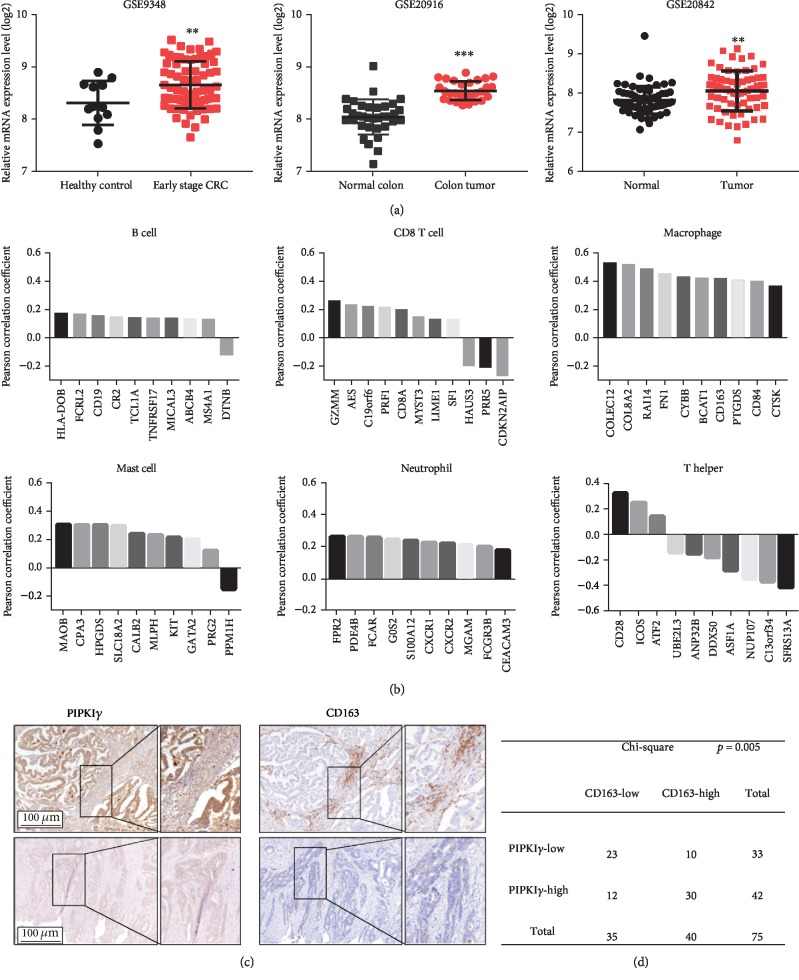
Increased PIPKI*γ* expression positively correlated with TAM infiltration in CRC. (a) mRNA expression levels of *PIP5K1C* in CRC tumor tissue and corresponding nontumor tissue. (b) Correlation between *PIP5K1C* and specific gene signatures of B cells, CD8^+^ T cells, macrophages, mast cells, neutrophils, and T helper cells. (c) Immunohistochemical analysis of PIPKI*γ* expression and CD163 in a human CRC tissue microarray. Representative high and low PIPKI*γ* expression images are shown in the left panel, and the corresponding CD163 staining results are shown in the right panel. (d) The statistical results for PIPKI*γ* and CD163 tissue scores for low and high staining determined in the CRC cohort. Scale bar: 100 *μ*m. ^∗^*p* < 0.05, ^∗∗^*p* < 0.01, ^∗∗∗^*p* < 0.001.

By analyzing Gene Expression Omnibus (GEO) datasets, we found that PIPKI*γ* expression was upregulated in CRC (Figure 1(a)). To characterize the potential immune cell components in the CRC tumor microenvironment affected by PIPKI*γ*, an immunome compendium was built using The Cancer Genome Atlas (TCGA) data from purified immune cell subsets [16, 17]. Both innate immune cells (mast cells, macrophages, and neutrophils) and acquired immune cells (B, T helper 1 (Th1) and CD8+ T), as well as cytotoxic cells, were used for investigation (Figure 1(b)). Notably, we observed that PIPKI*γ* was highly correlated with the gene signatures of macrophages. In contrast, no obvious correlation was found between the expression of PIPKI*γ* and other immune component-related genes (Figure 1(b)). To test whether PIPKI*γ* is associated with macrophage recruitment, immunohistochemistry was performed on CRC primary cancer serial sections. The results showed that high PIPKI*γ* expression samples had a stronger capacity to recruit CD163+ macrophages than their low expression counterparts, suggesting that PIPKI*γ* might facilitate macrophage infiltration in CRC (Figure 1(c)). Moreover, statistical analysis of this independent sample cohort also revealed a positive association between PIPKI*γ* expression and the number of CD163+ macrophages in CRC tissues (Figure 1(d)). Thus, PIPKI*γ* might exhibit a regulatory role in macrophage infiltration in CRC.

### 3.2. Upregulated PIP5I*γ* Increased CCL2 Expression in CRC Cancer Cells

**Figure 2:**
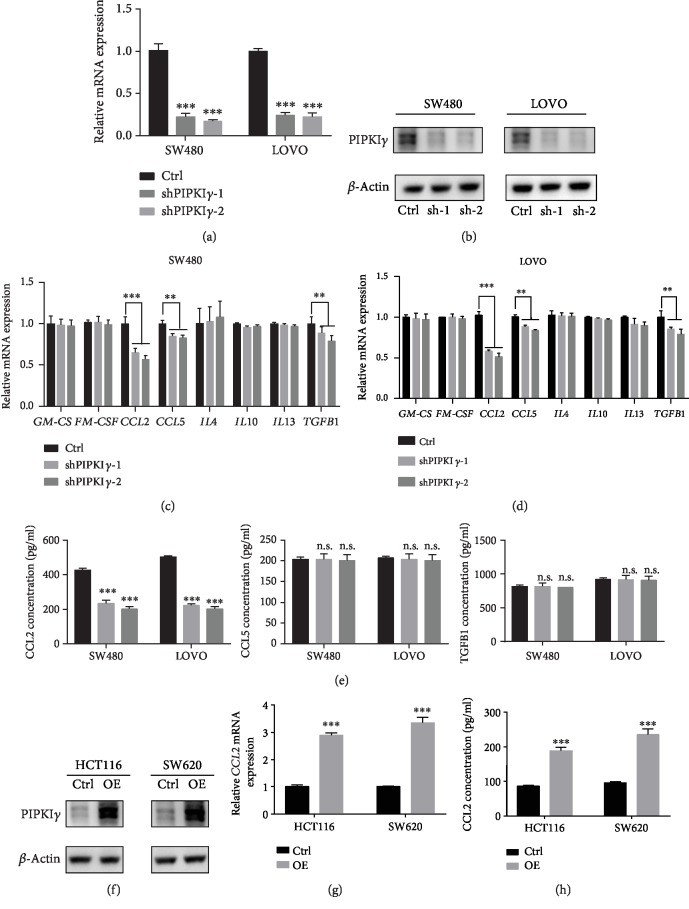
Upregulated PIP5I*γ* increased CCL2 expression in CRC cancer cells. (a) Stable shPIP5K1C-expressing SW480 and LOVO cells were grown in normal medium for 48 h, and PIP5K1C mRNA levels were measured by quantitative PCR with reverse transcription (RT-qPCR) (*n* = 3, data are the mean + SD) (b) Western blot analysis of the effect of PIPKI*γ* knockdown in SW480 and LOVO cells. Experiments were repeated twice, and representative results are presented. (c, d) Stable shPIP5K1C-expressing SW480 and LOVO cells were grown in normal medium for 48 h, and GM-CSF, M-CSF, CCL2, CCL5, IL4, IL10, IL13, and TGFB1 mRNA levels were measured by quantitative PCR with reverse transcription (RT-qPCR) (*n* = 3, data are the mean + SD). (e) Stable shPIP5K1C-expressing SW480 and LOVO cells were grown in FBS-free medium for 48 h, and ELISAs were performed to determine CCL2, CCL5, and TGFB1 levels in the CM from sh-Ctrl and sh-PIP5K1C SW480 and LOVO cells (*n* = 3, data are the mean + SD). (f) Western blot analysis of the effect of PIPKI*γ* overexpression in HCT116 and SW620 cells. Experiments were repeated twice, and representative results are presented. (g) q-PCR analysis of the mRNA level of CCL2 in PIPKI*γ*-overexpressing HCT116 and SW620 cells. (h) ELISA was performed to determine the protein level of CCL2 in PIPKI*γ*-overexpressing HCT116 and SW620 cells cultured in FBS-free medium.

To investigate whether PIP5I*γ* might enhance the recruitment of macrophages, we first examined the regulation of macrophage chemokines by PIP5I*γ*. SW480 and LOVO cells, two CRC cell lines with high PIP5I*γ* expression, were selected to construct stable knockdown cell lines. Q-PCR and immunoblot assays were performed to detect the efficiency of PIP5I*γ* silencing. The results showed that PIP5I*γ* expression was knocked down by more than 80% at both the mRNA and protein levels (Figures 2(a) and 2(b)). Next, we assessed the expression of macrophage chemokines in tumor cells harboring PIP5I*γ* short hairpin RNA (shRNA). Among these chemokines and cytokines, only CCL2 and CCL5 presented significantly downregulated. TGF*β*1 expression present lightly changed (Figures 2(c) and 2(d)). Consistent with the qPCR analysis of macrophage-related chemokines and cytokines, enzyme-linked immunosorbent assays (ELISAs) showed that CCL2 expression was greatly decreased in both SW480 and LOVO cell lines. However, CCL5 and TGF*β*1 remained almost unchanged (Figure 2(e)). To further characterize the regulation of CLL2 expression by PIP5I*γ*, PIP5K1C was transfected into two low PIP5I*γ* expression CRC cell lines (HCT116 and SW620) (Figure 2(f)). In agreement with the loss-of-function experiments, CCL2 expression in HCT116 and SW620 cells was remarkably increased upon PIP5K1C transfection (Figures 2(g) and 2(h)).

### 3.3. STAT3 Transcriptionally Regulates PIP5I*γ* to Increase CCL2 Expression

**Figure 3:**
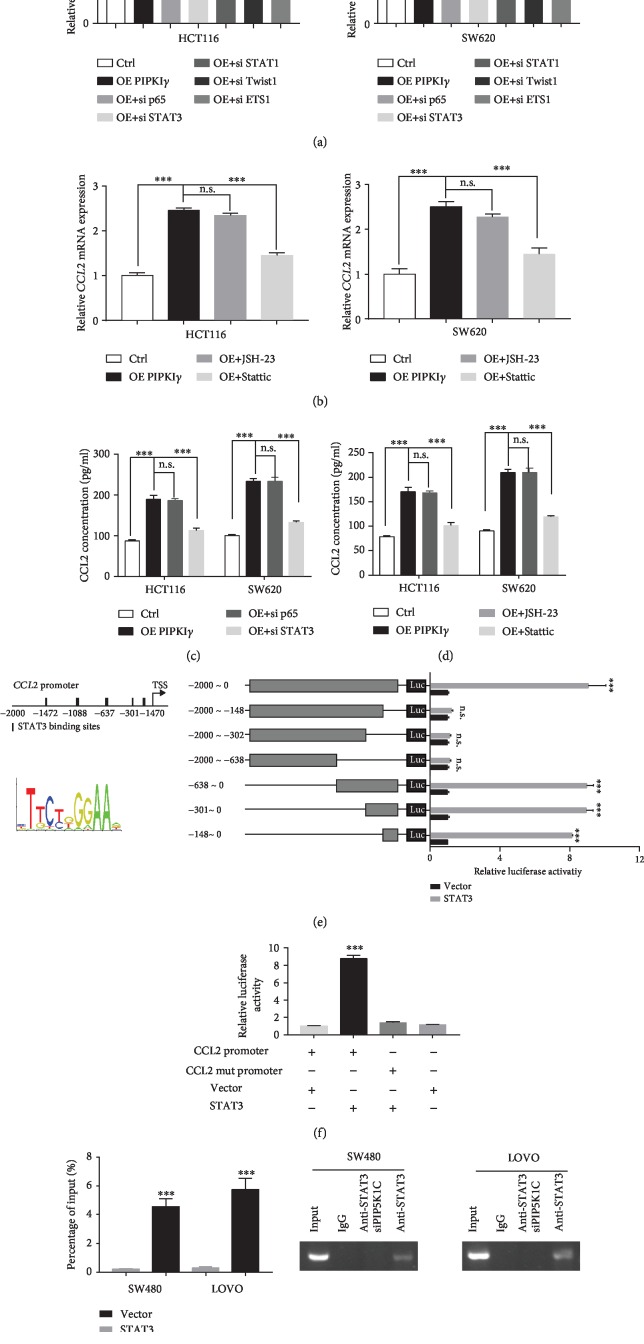
STAT3 transcriptionally regulates PIP5I*γ* to increase CCL2 expression. (a) Stable PIPKI*γ*-expressing HCT116 and SW620 cells were transfected with siP65, siSTAT3, siSTAT1, siTwist1, or siETS1 for 48 h, and the CCL2 mRNA level was measured by quantitative PCR with reverse transcription. (b) CCL2 mRNA expression levels were detected in stable PIPKI*γ*-expressing HCT116 and SW620 cells treated with JSH-23 and Stattic in FBS-free medium. (c) Stable PIPKI*γ*-expressing HCT116 and SW620 cells were transfected with si p65 and siSTAT3 for 48 h, and the CCL2 protein level was measured by ELISA. (d) CCL2 protein expression levels were detected in stable PIPKI*γ*-expressing HCT116 and SW620 cells treated with JSH-23 and Stattic in FBS-free medium. (e) The predicted STAT3 binding site on the upstream 0-2000 bp and the STAT3 binding motif. HEK 293 cells were transiently cotransfected with luciferase reporter plasmid (pGL3) containing the CCL2 DNA promoter region [-2000/0]) or different promoter fragment constructs, Renilla, vector, or STAT3 as indicated. After 30 h, luciferase activities were determined by dual luciferase assay. Luciferase activities were normalized to Renilla luciferase activities. The values indicated represent normalized luciferase activities and are shown as the mean ± S.E. from triplicate assays. (f) Luciferase assay for HEK 293 cells transiently cotransfected with luciferase reporter plasmid (pGL3) containing the CCL2 DNA promoter region [-2000/0]) or motif mutant constructs, Renilla, vector, or STAT3 as indicated. (g) ChIP-PCR analysis of STAT3 binding to the CCL2 promoter in the presence or absence of PIPKI*γ* silencing in SW480 and LOVO cells.

Next, we aimed to reveal the underlying molecular mechanism through which high PIP5I*γ* expression induces CCL2 upregulation. Considering that CCL2 mRNA expression increased upon ectopic expression of PIP5I*γ* in CRC cells, transcription factor regulation was considered a top candidate. Previous studies reported that CCL2 could be transactivated by NF-*κ*B, STAT3, STAT1, Twist1, and ETS1 [18–22]. Thus, we silenced these molecules in HCT116 and SW620 cells ectopically expressing PIP5I*γ*. The results showed that STAT1, Twist1, and ETS1 knockdown did not considerably affect CCL2 mRNA levels. However, siRNA-induced STAT3 depletion blocked the CCL2 increase induced by PIP5I*γ* (Figure 3(a)). The similar results were overserved in SW480 and LOVO (Supplementary [Supplementary-material supplementary-material-1]a). In addition, the results indicated that NF-*κ*B seems to be involved in CCL2 expression regulation. To further reveal one or both transcription factors involved in CCL2 transactivation, JSH-23 and Stattic, inhibitors of NF-*κ*B and STAT3, were administered to PIP5I*γ*-overexpressing CRC cells or high PIP5I*γ* expression CRC cells. The results showed that only Stattic treatment dramatically reduced CCL2 expression, and the NF-*κ*B inhibitor barely changed the CCL2 mRNA level (Figure 3(b) and Supplementary [Supplementary-material supplementary-material-1]b). Consistently, ELISA data also implied that only STAT3 was involved in CCL2 expression regulation (Figures 3(c) and 3(d) and Supplementary [Supplementary-material supplementary-material-1]c–d). To gain further evidence that CCL2 was directly transactivated by STAT3, we screened the potential binding site on the CCL2 promoter by performing a dual luciferase reporter gene assay. Among the five predicted sites, STAT3 was predominantly associated with the -147~-138 motif (Figure 3(e)), which was further confirmed by introducing mutations at this site. STAT3 transfection could not induce CCL2 upregulation after -147~-138 motif mutation (Figure 3(f)). To definitively prove that PIP5I*γ* induced CCL2 expression via STAT3, CHIP-PCR was performed on PIP5I*γ*-depleted SW480 and LOVO cells. As expected, CCL2 promoter binding to STAT3 was sharply reduced (Figure 3(g)). Taken together, these results suggested that STAT3 is the mediator between PIP5I*γ* and CCL2.

### 3.4. AKT Activation by PIPKI*γ* Mediated STAT3 Phosphorylation and CCL2 Expression in CRC

**Figure 4:**
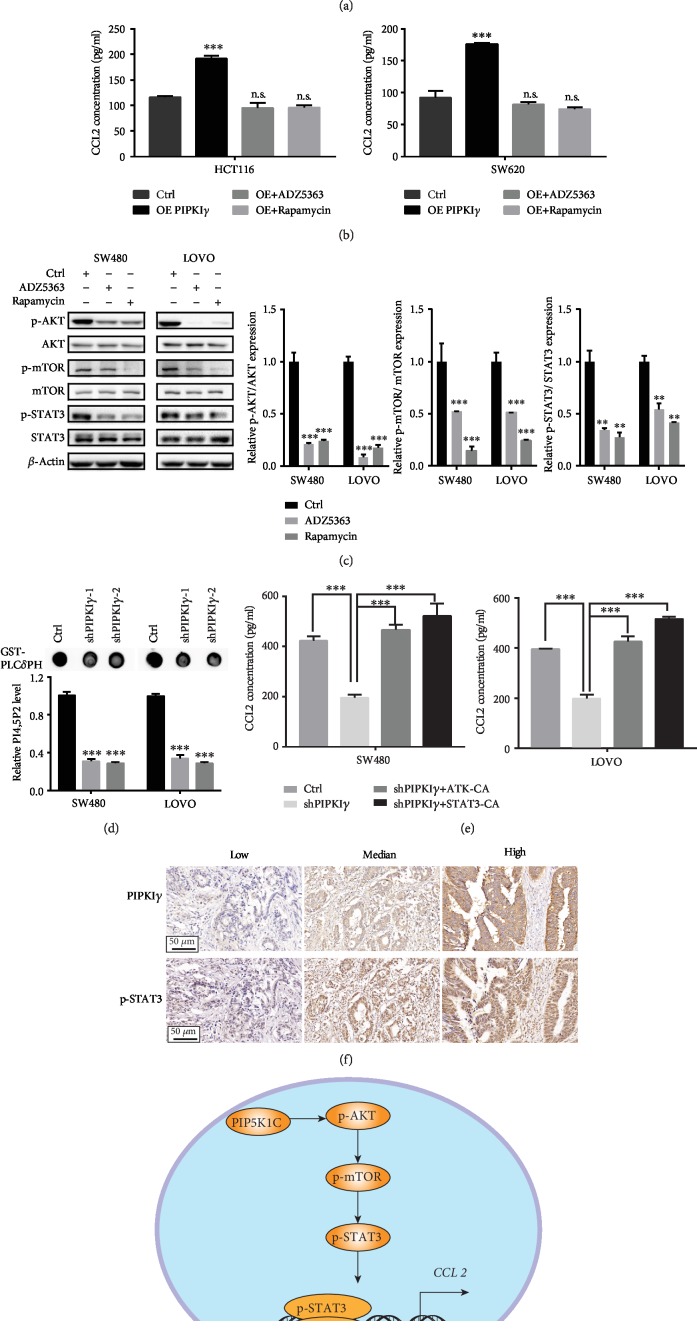
AKT activation by PIPKI*γ* mediated STAT3 phosphorylation and CCL2 expression in CRC. (a) q-PCR analysis of CCL2 mRNA levels in HCT116 and SW620 cells overexpressing PIPKI*γ* or not and treated with the AKT inhibitor ADZ5363 or the mTOR inhibitor rapamycin. (b) ELISA analysis of CCL2 protein levels in HCT116 and SW480 cells overexpressing PIPKI*γ* or not and treated with the AKT inhibitor ADZ5363 or the mTOR inhibitor rapamycin in FBS-free medium. (c) Western blot analysis of the phosphorylation levels of AKT, mTOR, and STAT3 in SW480 and LOVO cells treated with the AKT inhibitor ADZ5363 or the mTOR inhibitor rapamycin. Densitometric analysis presented in the right plane. Experiments were repeated twice; representative results are presented. (d) PIP2 level in the control, shPIPKI*γ*1, and shPIPKI*γ*2 cells was examined by a protein-lipid overlay assay. (e) ELISA analysis of CCL2 protein levels in PIPKI*γ*-depleted SW480 and LOVO cells transfected with continuously activated AKT or STAT3 plasmid. (f) Immunohistochemical analysis of PIPKI*γ* and p-STAT3 expression in a human Renji CRC tissue microarray. Representative low, median, and high PIPKI*γ* expression images are shown in the upper panel, and the corresponding samples' p-STAT3 expression levels are shown in the lower panel. (g) Proposed mechanism of PIPKI*γ*-AKT-mTOR-STAT3-driven CCL2 expression in colorectal cancer cells.

Next, we aimed to elucidate the signaling pathway between PIPKI*γ* and STAT3. Considering that PIPKI*γ* functions as a crucial substrate of the PI3K/AKT pathway, we hypothesized that AKT might mediate the response to PIPKI*γ* and induce STAT3 phosphorylation. To verify this hypothesis, a specific inhibitor of AKT, ADZ5363, and a specific inhibitor of mTOR, rapamycin, were administered to PIPKI*γ*-overexpressing HCT116 and SW620 cells as well as SW480 and LOVO cells. The results showed that AKT and mTOR inhibition significantly blocked the increase in CCL2 induced by PIPKI*γ* overexpression (Figure 4(a) and Supplementary [Supplementary-material supplementary-material-1]), which was further confirmed by ELISA (Figure 4(b) and Supplementary [Supplementary-material supplementary-material-1]). To further confirm the necessity of the AKT-mTOR pathway in the interaction between PIPKI*γ* and STAT3, AKT and mTOR inhibitors were used to treat SW480 and LOVO cells. The phosphorylation levels of STAT3, AKT, and mTOR were detected in SW480 and LOVO cells upon ADZ5363 or rapamycin treatment. We noticed that the phosphorylation level of STAT3 was markedly reduced after AKT or mTOR suppression (Figure 4(c)). Considering that PIP_2_ is predominantly product of PIPKI*γ* and the precursor compound of PIP_3_, we measured the level of PIP_2_ and PIP_3_ in PIPKI*γ*-deficient CRC cells by protein-lipid overlay assay [23]. We observed that the level of PIP_2_ and PIP_3_ were significantly decreased in PIPKI*γ* knockdown cells (Figure 4(d) and Supplementary [Supplementary-material supplementary-material-1]). This indicates that PIP_2_ generated by PIPKI*γ* is associated with PIP_3_ synthesis, resulting in PI3K/Akt activation. Next, to prove that AKT and STAT3 are indeed involved in the PIPKI*γ*-induced CCL2 increase, continuously activated AKT and STAT3 were transfected into PIPKI*γ*-depleted SW480 and LOVO cells. The results indicated that both AKT and STAT3 could rescue the reduction in CCL2 (Figure 4(e) and Supplementary [Supplementary-material supplementary-material-1]). Additionally, PIPKI*γ* expression and p-STAT3 expression in CRC clinical samples were positively associated (Figure 4(f)). Collectively, these results suggested that PIPKI*γ* may activate the PI3K-Akt-STAT3 signaling pathway, which can further increase CCL2 levels in colorectal cancer (Figure 4(g)).

## 4. Discussion

Although early detection and overall survival have improved, colorectal cancer (CRC) remains one of the leading causes of death worldwide. Current treatment paradigms, including chemotherapy and biologics, appear to have hit their bottleneck. Immunotherapy, especially checkpoint inhibitors, has shown considerable clinical benefits in a variety of cancers, including CRC. However, previous studies reported that only some patients respond to checkpoint inhibitors [24, 25], emphasizing the importance of further understanding the mechanism of immunosuppression microenvironment formation. In this study, we found that PIPKI*γ* expression positively correlated with macrophage infiltration in colorectal cancer. Further studies revealed that high expression PIPKI*γ* could induce the AKT-mTOR pathway, leading to increased STAT3 phosphorylation and ultimately promoting CCL2 expression.

PIPKI*γ* functions mainly as a phosphoinositide-producing enzyme and is highly involved in phosphoinositide metabolism. Upregulation of PIPKI*γ* expression is often reported in human primary tumors. We have previously shown that PIPKI*γ* is upregulated in pancreatic cancer cell lines, indicating the pathogenic role of PIPKI*γ* in malignant transformation [15]. Additionally, pY639-PIPKI*γ* is significantly increased in invasive ductal carcinoma, suggesting the importance of PIPKI*γ* in tumor progression [26]. Through the generation of PI(4,5)P2, PIPKI*γ* is critically important in a variety of biological processes, such as focal adhesion assembly [27, 28], ciliogenesis [29], centriole duplication [30], and leukocyte recruitment [31]. Notably, PIPKI*γ* is also widely implicated in many oncogenic phenotypes, such as cell proliferation [10, 23], migration [32], invasion [26], and epithelial-to-mesenchymal transition [11]. Analysis of TCGA cohorts showed that increased PIPKI*γ* expression levels positively correlated with increased macrophage infiltration, indicating that PIPKI*γ* might act as a new modulator of the immunosuppression environment in colorectal cancer. By both gain- and loss-of-function studies, we confirmed that PIPKI*γ* could significantly regulate CCL2 at both the mRNA and protein levels. However, PIPKI*γ* had no significant impact on other monocyte-recruited cytokines or macrophage-induced cytokines. The effect of other immune cell-related cytokines should be further studied.

The PI3K/Akt signaling pathways are often activated in human cancers [33]. These pathways are initiated by the generation of PI [3–5] P3 via the PI3K-mediated phosphorylation of PI [4, 5] P2. Previously, Thapa et al. clearly demonstrated the mechanism by which PIPKI*γ* couples with PI3K to activate PI3K/Akt signaling [23]. In line with this, our results showed that the inhibition of p-Akt and downstream p-mTOR blocks PIPKI*γ*-induced STAT3 phosphorylation. Notably, PI3K/Akt signaling and the downstream mTORC1 complex are crucial regulators of STAT3 [34, 35]. In breast cancer, the PTEN/mTOR/STAT3 pathway plays a crucial role in cancer stem-like cell viability and stem maintenance [36]. In leukemia, blocking AKT-mTOR-STAT3 signaling through glycyrrhizic acid could remarkably inhibit leukemia cell migration and invasion [37]. Our data showed that high PIPKI*γ* expression induced AKT-mTOR-STAT3 signaling, leading to increased CCL2 transcription and immunosuppression microenvironment formation. Previous research has reported that STAT1, Twist1, ETS1, and NF-*κ*B are also involved in CCL2 transcription [18]. In our experiment, CCL2 mRNA and protein levels were decreased in only STAT3-disrupted CRC. Similarly, the regulation of CCL2 by STAT3 was also reported in prostate cancer cells and cancer-associated fibroblasts [20, 38].

The infiltration of tumor-associated macrophages (TAMs) has been linked to tumor progression in many tumor diseases, suggesting the potential of TAM-targeting therapy for the treatment of advanced cancer [39]. It has been shown that TAMs can provide growth and survival factors, induce angiogenesis [40, 41], and enhance matrix remodeling [42] in the tumor microenvironment, consequently leading to tumor metastases. On the other hand, TAMs are a major component of immunosuppressive cells in almost all types of tumors. TAMs express molecular inducers of checkpoint proteins that inhibit T cell activation. TAMs promote the immunosuppressive activity of regulatory T (Treg) cells via bidirectional interactions, which are mediated by immunosuppressive cytokines, including IL-10 and TGF*β*. Moreover, many essential amino acids are consumed by TAMs, resulting in the metabolic starvation of T cells.

CCL2, also known as MCP1, binds to CCR2 and mediates LY6C^hi^ monocyte recruitment [43]. However, the precise mechanism by which CCL2 recruit monocytes/macrophages remains unclear. One potential mechanism is that circulating CCL2 associates with glycosaminoglycans in specific tissues and establishes gradients that guide monocytes towards these sites [44]. There is sufficient evidence that CCL2 recruits monocytes and macrophages in the tumor microenvironment [45–47]. CCL2 is highly expressed in malignant tumor cells and may play an essential role in TAM recruitment [48, 49]. After recruitment, TAMs can also produce CCL2, suggesting that a positive feedback loop may exist in the crosstalk between cancer cells and TAMs. In addition, CCL5 is another typical chemokine that recruits monocytes. In our study, we found that its mRNA expression, but not its protein expression, fluctuated after disrupting PIPKI*γ* expression. Considering that CCL5 has been reported to coordinate with the *β*-catenin/Slug pathway to promote CRC epithelial-mesenchymal transition (EMT) [50], we speculated that CRC cells may operate via another compensatory pathway to promote CCL5 translation. Besides, Chun et al. reported that CCL2 fostered MDSC accumulation in evolving colonic tumors and enhanced polymorphonuclear- (PMN-) MDSC immunosuppressive features, leading to the progression of colorectal cancer [51]. Thus, CCL2 may also promote other immune suppression cell functions, which may require more effort.

Collectively, our observations revealed that high PIPKI*γ* expression in tumor cells could induce AKT-mTOR signaling activation. Increased activation of AKT-mTOR increases the phosphorylation levels of STAT3, leading to CCL2 expression and suppressing cancer immune reaction activation. These findings lay the theoretical foundation for targeting PIPKI*γ* for the treatment of colorectal cancer patients.

## Data Availability

All data that support the findings of this study are available from the authors upon reasonable request.

## Supplementary Materials

Supplementary 1 Supplementary Figure 1: (a) SW480 and LOVO cells were transfected with siP65, siSTAT3, siSTAT1, siTwist1, or siETS1 for 48 h, and the CCL2 mRNA level was measured by quantitative PCR with reverse transcription. (b) CCL2 mRNA expression levels were detected in SW480 and LOVO cells treated with JSH-23 and Stattic in FBS-free medium. (c) S SW480 and LOVO cells were transfected with si p65 and siSTAT3 for 48 h, and the CCL2 protein level was measured by ELISA. (d) CCL2 protein expression levels were detected in SW480 and LOVO cells treated with JSH-23 and Stattic in FBS-free medium. Supplementary Figure 2: (a) q-PCR analysis of CCL2 mRNA levels in SW480 and LOVO cells treated with the AKT inhibitor ADZ5363 or the mTOR inhibitor rapamycin. (b) ELISA analysis of CCL2 protein levels in H SW480 and LOVO cells treated with the AKT inhibitor ADZ5363 or the mTOR inhibitor rapamycin in FBS-free medium. (c) PIP3 level in the control, shPIPKI*γ*1, and shPIPKI*γ*2 cells was examined by a protein-lipid overlay assay. (d) ELISA analysis of CCL2 protein levels in HCT116 and SW620 transfected with continuously activated AKT or STAT3 plasmid.

Supplementary 2 Supplementary Material Table 1 is “Q-PCR primers used in this study”.
