# Corrigendum to “PIPKI*γ* Regulates CCL2 Expression in Colorectal Cancer by Activating AKT-STAT3 Signaling”

**DOI:** 10.1155/2020/1786505

**Published:** 2020-09-30

**Authors:** JunLi Xue, XiaoXiao Ge, Wei Zhao, Liqiong Xue, Congqi Dai, Fengjuan Lin, Wei Peng

**Affiliations:** Department of Oncology, Shanghai East Hospital, Tongji University School of Medicine, 1800 Yuntai Road, Pudong District, Shanghai 200123, China

In the article titled “PIPKI*γ* Regulates CCL2 Expression in Colorectal Cancer by Activating AKT-STAT3 Signaling” [1], the Acknowledgments section should read as follows:
