# Supplementary material for: PIPKIγ Regulates CCL2 Expression in Colorectal Cancer by Activating AKT-STAT3 Signaling
Source: J Immunol Res. 2019 Nov 3;2019:3690561. doi: 10.1155/2019/3690561 (PMC6874988; doi:10.1155/2019/3690561)
Supplement: Supplementary 1 — Supplementary Figure 1: (a) SW480 and LOVO cells were transfected with siP65, siSTAT3, siSTAT1, siTwist1, or siETS1 for 48 h, and the CCL2 mRNA level was measured by quantitative PCR with reverse transcription. (b) CCL2 mRNA expression levels were detected in SW480 and LOVO cells treated with JSH-23 and Stattic in FBS-free medium. (c) S SW480 and LOVO cells were transfected with si p65 and siSTAT3 for 48 h, and the CCL2 protein level was measured by ELISA. (d) CCL2 protein expression levels were detected in SW480 and LOVO cells treated with JSH-23 and Stattic in FBS-free medium. Supplementary Figure 2: (a) q-PCR analysis of CCL2 mRNA levels in SW480 and LOVO cells treated with the AKT inhibitor ADZ5363 or the mTOR inhibitor rapamycin. (b) ELISA analysis of CCL2 protein levels in H SW480 and LOVO cells treated with the AKT inhibitor ADZ5363 or the mTOR inhibitor rapamycin in FBS-free medium. (c) PIP3 level in the control, shPIPKIγ1, and shPIPKIγ2 cells was examined by a protein-lipid overlay assay. (d) ELISA analysis of CCL2 protein levels in HCT116 and SW620 transfected with continuously activated AKT or STAT3 plasmid. [file 3690561.f1.docx]

**Supplementary methods**

**RT-PCR**

Total RNA was extracted from in vitro indicated cells using Qiagen RNeasy kits (Qiagen Valencia, CA). Next, complementary DNA was synthesized using a First Strand cDNA Synthesis Kit (ThermoFisher Scientific) at 37℃ for 60 min. Subsequently, cDNA was subjected to PCR amplification on ABI7500 real-time System (Applied Biosystems) to analyze the expression of mRNA. Comparative 2–ΔΔCt method applied for analysis the fold change in mRNA level between treatment groups. 18S RNA was used as an internal control. The detail information of primers used in this study listed in supplementary Table 1.

**Western immunoblots**

Cells were washed twice with PBS and were then lysed in lysis buffer supplemented with protease and phosphatase inhibitors (Thermo Scientific, 1861281). Cell lysates concentrations were measured by Bio-Rad Protein Assay Reagent depending on the manufacturer’s instructions. Subsequently, cell lysates were separated by 10%-12% SDS-PAGE and then electrophoretically transferred onto PVDF membranes. 5% defatted milk was used for membranes blocking. After that, the blocked membranes were incubated with indicated primary antibodies overnight at 4 °C, and washed three times with TBST, followed by incubation with corresponding secondary antibodies for 2hr and washed three times with TBST. β-actin antibody was selected as loading control. Immunoblots were captured using the Pierce™ Western ECL Blotting substrate (ThermoFisher Scientific, Waltham, MA) and ChemiDoc Touch image system (Bio-Rad).The antibodies used were listed as follows: PIPKIγ (1: 2,000, Abcam, ab109192), p-Akt (1:2,000, Cell Signaling Technology, #4060), Akt (1:1,000, Cell Signaling Technology, #4685), p-mTOR (1:1,000, Cell Signaling Technology, #2971), mTOR (1:1,000, Cell Signaling Technology, #2983), p-STAT3 (1:1,000, Cell Signaling Technology, #9145), STAT3 (1:1,000, Cell Signaling Technology, #9139), and β-actin (1:1,000, Abcam, ab8227).

**Protein-Lipid Overlay Assay**

PIP2 and PIP3 were detected by a protein-lipid overlay assay. Briefly, acidic lipids containing PIP2 and PIP3 were isolated from control or stably shPIPKIγ expression CRC cells following the protocolprovided by Echelon Biosciences. Equal numbers of cells were used for the lipid extraction Isolated lipids were dissolved into MeOH-CHCl_3_-HCland spotted onto nitrocellulose membrane, followed by blocking with 3% BSA in Tris-buffered saline (TBS) containing 0.1%Tween 20 (TBS-T). Membranes were incubated with GSTPLC-PH (0.5 g/ml; Echelon Biosciences) or GST-GRP1-PH
(1 g/ml; Echelon Biosciences) overnight at 4 °C. The bound proteins with the lipids in the membrane were detected using HRP-labeled anti-GST antibody (Sigma).

**Supplementary figures**

Supplementary Figure 1


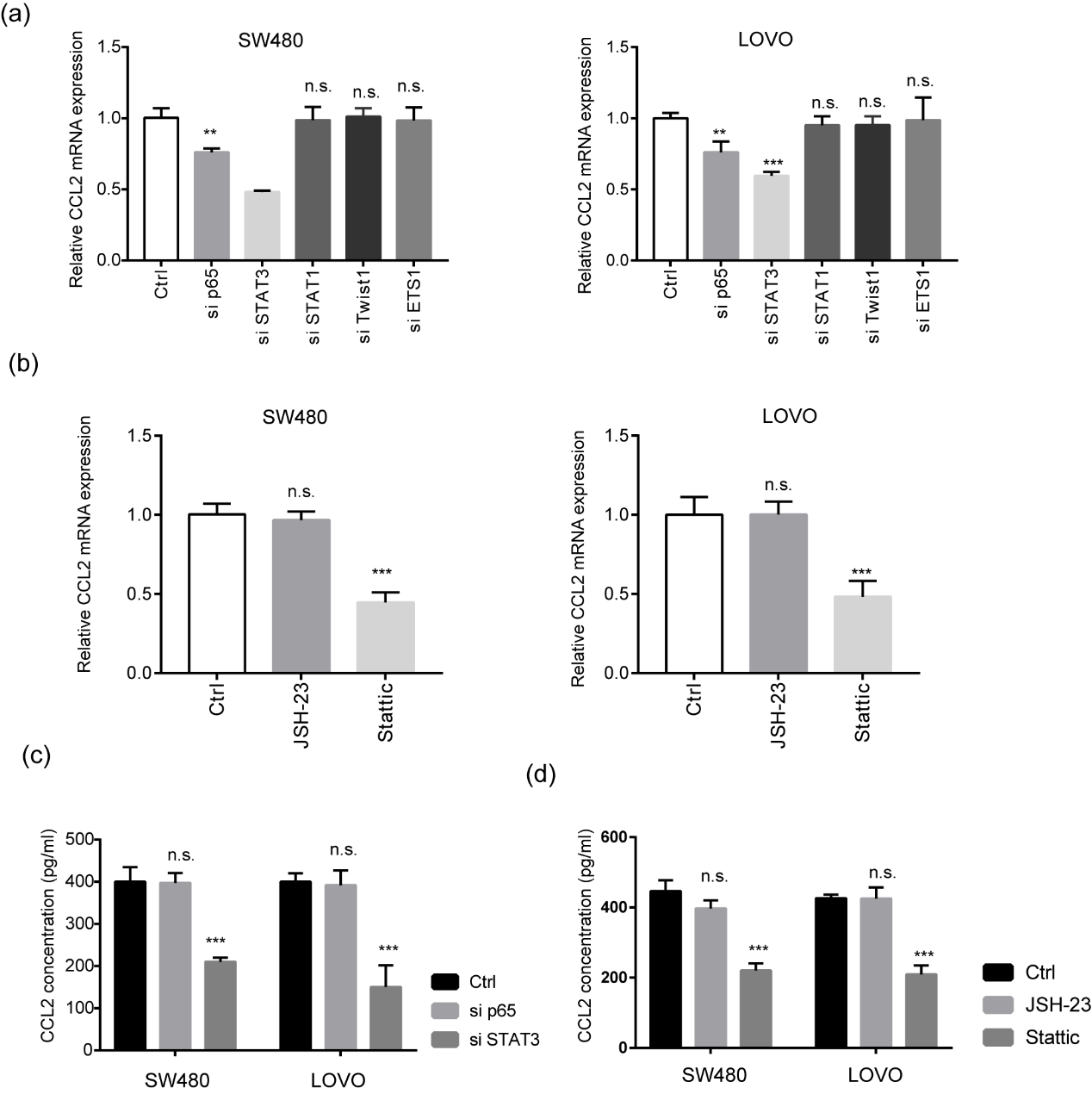


Supplementary Figure 1.

(a) SW480 and LOVO cells were transfected with siP65, siSTAT3, siSTAT1, siTwist1 or siETS1 for 48 h, and the CCL2 mRNA level was measured by quantitative PCR with reverse transcription. (b) CCL2 mRNA expression levels were detected in SW480 and LOVO cells treated with JSH-23 and Stattic in FBS-free medium. (c) S SW480 and LOVO cells were transfected with si p65 and siSTAT3 for 48 h, and the CCL2 protein level was measured by ELISA. (d) CCL2 protein expression levels were detected in SW480 and LOVO cells treated with JSH-23 and Stattic in FBS-free medium.

Supplementary Figure 2.


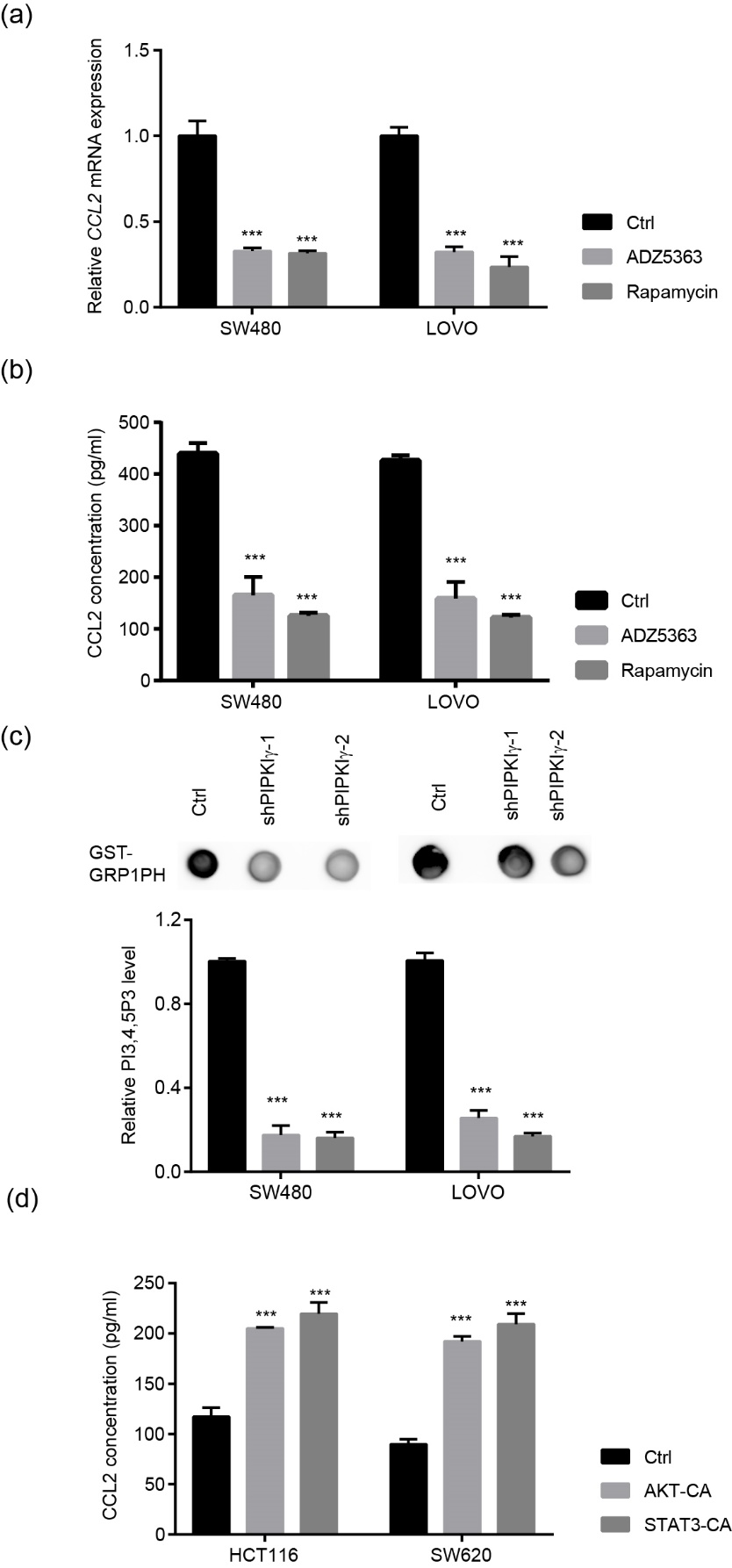


Supplementary Figure 2.

(a) q-PCR analysis of CCL2 mRNA levels in SW480 and LOVO cells treated with the AKT inhibitor ADZ5363 or the mTOR inhibitor rapamycin. (b) ELISA analysis of CCL2 protein levels in H SW480 and LOVO cells treated with the AKT inhibitor ADZ5363 or the mTOR inhibitor rapamycin in FBS-free medium. (c) PIP_3_ level in the control, shPIPKIγ1 and shPIPKIγ2 cells was examined by a protein-lipid overlay assay. (d) ELISA analysis of CCL2 protein levels in HCT116 and SW620 transfected with continuously activated AKT or STAT3 plasmid.
